# The Significance of COVID-19 Diseases in Lipid Metabolism Pregnancy Women and Newborns

**DOI:** 10.3390/ijms232315098

**Published:** 2022-12-01

**Authors:** Miljana Z. Jovandaric, Milan Dokic, Ivana R. Babovic, Srboljub Milicevic, Jelena Dotlic, Branislav Milosevic, Miljan Culjic, Luka Andric, Nemanja Dimic, Olga Mitrovic, Aleksandra Beleslin, Jovana Nikolic, Zorica Jestrovic, Sandra Babic

**Affiliations:** 1 Department of Neonatology, Clinic for Gynecology and Obstetrics, University Clinical Center of Serbia, 11000 Belgrade, Serbia; 2 Department of Gynecology and Obstetrics, Clinic for Gynecology and Obstetrics, University Clinical Center of Serbia, 11000 Belgrade, Serbia; 3 Medical Faculty, University of Belgrade, 11000 Belgrade, Serbia; 4 Clinic for Anesthesiology and Intensive Care, University Clinical Hospital Center “Dr Dragisa Misovic - DEDINJE”, 11000 Belgrade, Serbia

**Keywords:** COVID-19, pregnancy, newborn, lipid peroxidation

## Abstract

Coronavirus disease (COVID-19) is an infectious disease caused by SARS-CoV-2. Elderly people, people with immunodeficiency, autoimmune and malignant diseases, as well as people with chronic diseases have a higher risk of developing more severe forms of the disease. Pregnant women and children can becomesick, although more often they are only the carriers of the virus. Recent studies have indicated that infants can also be infected by SARS-CoV-2 and develop a severe form of the disease with a fatal outcome. Acute Respiratory Distress Syndrome (ARDS) ina pregnant woman can affect the supply of oxygen to the fetus and initiate the mechanism of metabolic disorders of the fetus and newborn caused by asphyxia. The initial metabolic response of the newborn to the lack of oxygen in the tissues is the activation of anaerobic glycolysis in the tissues and an increase in the concentration of lactate and ketones. Lipid peroxidation, especially in nerve cells, is catalyzed by iron released from hemoglobin, transferrin and ferritin, whose release is induced by tissue acidosis and free oxygen radicals. Ferroptosis-inducing factors can directly or indirectly affect glutathione peroxidase through various pathways, resulting in a decrease in the antioxidant capacity and accumulation of lipid reactive oxygen species (ROS) in the cells, ultimately leading to oxidative cell stress, and finally, death. Conclusion: damage to the mitochondria as a result of lipid peroxidation caused by the COVID-19 disease can cause the death of a newborn and pregnant women as well as short time and long-time sequelae.

## 1. Introduction

Human coronaviruses (HCoVs) periodically emerge around the world. They can be extremely pathogenic to humans, such as severe acute respiratory syndrome coronavirus-2 (SARS-CoV-2)—a disease called COVID-19 [1]. The SARS-CoV-2 virus consists of four structural proteins: spike (S), envelope protein (E), membrane (M) and nucleocapsid (N). The receptor binding domain (RBD) is responsible for the binding of the virus to ACE2. The S protein gives the appearance of a crown (corona); it has two subunits, the S1 subunit binds to the receptor, and the S2 subunit binds the S protein to the cell membrane and joins the cell.

Angiotensin-converting enzyme 2 (ACE2) is an important regulator of the renin-angiotensin system (RAS), and SARS-CoV-2 is transmitted by binding to ACE2 receptors in the cells of various tissues. ACE2 is mainly found in the endothelium, lungs, heart, kidneys and intestine, which coincides with the finding of SARS-CoV-2 in multiple organs. The viruses are able to alter the host’s lipid metabolism to enhance their replication capacity within the infected host cell, which is accompanied by increased production of inflammatory mediators. SARS-CoV-2 infection increased the expression of key proteins in the regulation of lipid metabolism and the amount of LD per cell. Changes in the lipid metabolism in the cells and plasma appear as major phenotypes during an infection with COVID-19 and SARS-CoV-2 [2].The patients infected with SARS-CoV-2 in most of the cases have symptoms associated with pneumonia, including fever, cough, myalgia or fatigue, however, an infection with SARS-CoV-2 can lead to symptoms of the diseases associated with other tissues, which are digestive ones (diarrhea, poor appetite, nausea and vomiting), those in the nervous system (confusion and headache) and cardiovascular ones (chest pain and myocarditis) [3].

The ACE2 expression levels are highest in the small intestine, testis, kidneys, heart, thyroid, and adipose tissue, and they are the lowest in the blood, spleen, bone marrow, brain, blood vessels, and muscle. The predominant symptoms of patients infected with SARS-CoV-2 could be attributed to the fact that the respiratory tract is the readiest transmission approach for the virus [4].

Epidemiologic studies highlight that elderly people, people with immunodeficiency, autoimmune and malignant diseases, as well as people with chronic diseases (such as chronic hypertension, heart failure, diabetes, liver disorders and respiratory diseases) have a higher risk of developing more severe forms of the disease [5].

Pregnant women and children can also become infected, although more often they are only the carriers of the virus. Recent studies have indicated that infants can also be infected withSARS-CoV-2 and develop a severe form of the disease with a fatal outcome [6].

### COVID-19 in Pregnancy

**Figure 1:**
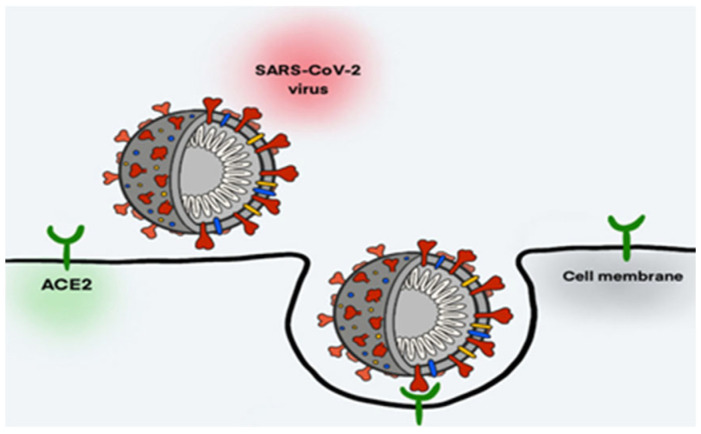
Diagram of SARS-CoV-2 entry into host cell. The spike glycoprotein (red), which consists of two subunits, binds to the ACE2 receptor (green) of host cells to merge the viral and cellular membranes and insert the viral genomic RNA (white) into the host cell. This induces endocytosis and the merging of the viral and cellular membranes, causing the viral genomic RNA to be inserted into the host cell, and thus, allowing the virus to replicate. The binding of SARS-CoV-2 to the ACE2 receptor causes a downregulation of this receptor, disrupting its normal function in maintaining immune homeostasis and leading to pro-inflammatory effects that can cause lung injury. The figure was reproduced from [8] (Atzrodt CL et al., 2020).

An important aspect that makes pregnant women more susceptible to COVID-19 is the fact that SARS-CoV-2 uses an enzyme that converts angiotensin ACE2 intoa receptor for cell invasion. ACE2 acts as a gateway for the virus in the placenta and fetal circulation [7]. An examination of the placental tissue for the presence and localization of SARS-CoV-2 spike glycoprotein (CoV2 SP) has revealed that this viral protein is present in the villous compartment of all of the placentas in pregnant women who tested positive for COVID-19 [8] (Figure 1).

A SARS-CoV-2 placental infection has been proven during pregnancy. Placental blood vessels, as well as the placental tissue itself, are similar to the lung tissue, and the SARS-CoV-2 virus S protein binds to the placental blood vessels via the ACE2 receptor. The studies of the placenta and fetal organs in early pregnancy abortion in pregnant women infected with the SARS-CoV-2 virus proved the existence of viral RNA in the placenta and fetal tissue [9].

Debelenko et al. observed syncytiotrophoblast (STB) damage which lead to placental trophoblastic necrosis accompanied by intervillosis and perivillous fibrin deposition in the placenta of COVID-19-positive women [10]. The is similar to Debenko et al.’s findings of trophoblastic necrosis and it is supportedby Garrido-Pontnou et al.’s findings that about 4.5% of the placentas collected from women with COVID-19 showed similar features of trophoblast necrosis, as well as, intervillous space collapse [11]. Trophoblastic necrosis with inflammatory infiltration is a characteristic of anSARS-CoV-2 infection of the placenta, and this is associated with fetal demise [10].

## 2. COVID-19 Diseases in Lipid Metabolism Pregnancy Women

### 2.1. Metabolic Changes in Non-COVID-Positive Pregnant Women

Pregnancy involves anatomical, physiological and biochemical adaptations that are important to provide proper fetal nutrition [12].

Carbohydrate metabolism in pregnancy is complex and not fully understood. The resultsare, primarily, changes in the hormonal balance during pregnancy and the formation of a new “glucose feto-placental shunt”. On the other hand, during pregnancy, the body’s response to glucose load changes, i.e., an insulin resistance appears, most likely under the influence of resistin (Retn) is a pro-inflammatory adipokine, which has been identified as an “adipose-tissue-specific secretory factor” (ADSF), and it was eventually renamed “resistin” (or “resistance to insulin”) [13].

The secretion of hormones that affect glucose metabolism changes qualitatively and quantitatively during pregnancy. In addition to these factors, the metabolism is influenced by the placenta through the production of estrogen, progesterone and hPL. The main energy substance for the fetus is glucose, which passes the placental barrier by facilitated diffusion [14].

At the beginning of the pregnancy, we find hypoglycemia with hypoinsulinemia and an increased turnover of amino acids. This is explained by intensive processes of glycogenesis and lipogenesis, with the accumulation of subcutaneous fatty tissue inpregnant women. Adiponectin is secreted by adipocytes, and it has multiple roles in the stimulation of lipid metabolism, glucose uptake, it displays anti-inflammatory properties and it correlates inversely with body weight and fat mass. During pregnancy, the adiponectin concentration drops due to an increase in the fat mass, and it has been negatively correlated with birth weight, suggesting that adiponectin may be involved in placental nutrient transport [15].

Leptin is secreted by fat cells and the placenta, and it participates in regulating food intake, energy homeostasis, insulin secretion, as well as the transport of nutrients to the fetus [16]. The amount of insulin increases during pregnancy. The most important diabetogenic effect in pregnancy is hPL, which is biochemically similar to the growth hormone and whose production increases during pregnancy, reducing the oxidation of glucose in the cell. This hormone acts anabolically on the proteins and lipolytically on the fats (increases the concentration of free fatty acids), despite increased lipogenesis. hPL is produced independently of glycemia, in contrast to growth hormone, whose production decreases with hyperglycemia, and vice versa [17].

Cholesterol and triacylglycerol decrease most often in the first seven weeks of pregnancy, and then, they increase until the end of pregnancy. There is a hypothesis that the increase in the concentration of free fatty acids (FFA) in the blood of pregnant women duringadvanced pregnancy is the result of a decrease in the sensitivity of insulin to the concentration of glucose in the blood. In pregnant women, there is a physiological increase in their body weight and a change in the fat metabolism [18].

Excessiveadipose tissue AT accumulation is characterized by the dysregulation of adipokine release, including leptin, adiponectin, and resistin [19].Excessive AT generates an imbalance between the prooxidative, and antioxidative systems that usually results in anincrease in the number of reactive oxygen species (ROS) [20]. While low ROS levels are essential to maintaining diverse physiological functions, excessive ROS production alters different cellular components such as proteins, lipids and DNA, generating oxidized biomolecules that function as the biomarkers of oxidative damage. The elevation of malondialdehyde (MDA), carbonylated proteins (CP) and oxidized base 8-oxo-2′-deoxyguanosine (8-oxodG) as indicators of lipid peroxidation, protein and DNA oxidation, respectively, hasdeleterious effects on the cells [21].

### 2.2. The Relation of SARS-CoV-2 to Respiratory and Metabolic Changes in Pregnancy

During pregnancy, the symptoms of COVID-19 are similar to the symptoms of it in non-pregnant women. Severe pneumonia occurred in from 0 to 14% of them [22]. Acute respiratory distress syndrome (ARDS) occurred when fluid built up in the alveoli of the lungs.Accumulated fluid in the alveoli prevents proper breathing and the supply of air to the lungs, and thus, insufficient oxygenation of the blood stream. In this way, the complete hypoxia of all of the organs occurs, especially in the heart and brain. ARDS occurs most often in pregnant women who have comorbidities (diabetes mellitus, chronic hypertension, chronic kidney disease, cardiovascular disease and previous brain and heart infarctions) usually within a few hours or days of the infection [23]. The respiratory distress syndrome ina pregnant woman can affect the supply of oxygen to the fetus and initiate the mechanism of metabolic disorders of the fetus and newborns, which are caused by asphyxia [24]. In the absence of oxygen in utero in the placenta, the microcirculation is disturbed, which causes a metabolic disorder in the mother–fetus system because there is a disturbance in the exchange of nutrients, and later, also a disturbance in the transport of oxygen. Oxidative stress (OA) is defined as the excessive formation and/or insufficient removal of free radicals and their products due to an impairment of the anti-oxidative (AO) capacities. As a consequence, proteins, DNA, lipids and other biomolecules are damaged, which leads to pathological changes in the body. Protein oxidation can be twofold: the free radicals can act directly on the protein or indirectly as a result of the oxidation of other molecules (lipids or carbohydrates). The oxidative modification of DNA is most pronounced in the presence of a metal that has a variable valence, and then, OH• is formed as a product of the oxidative reaction. The resulting radical reacts with the purine base guanine, and 8-hydroxydeoxyguanosine (8-OHdG) is formed, which is an indicator of oxidative DNA damage [19]. Leukotrienes and prostaglandins formed from arachidonic acid and free oxygen radicals increasethe microvascular permeability, which leads to the transendothelial migration of leukocytes and their infiltration into ischemic brain tissue and the activation of the inflammatory reaction. The result is the further release of a large amount of oxygen free radicals (OFR): superoxide anions, hydrogen peroxide and hydroxyl radicals. OFRs worsen the tissue damage and biochemical functioning of the cells, causing the peroxidation of the lipid component of the cell membrane and a further effect on the cell lipids, proteins and nucleic acids, which enables the invasion and infiltration of neutrophils into the post-ischemic brain tissue. NO synthetase increases the NO concentration, and an abnormally high amount of superoxide converts NO to peroxynitrite, which damages the capillary endothelium and increases not only edema, but also leads to endothelial protrusions that further block the capillaries. Neuroinflammation and oxidative stress (OS) can initiate a cascade of events, leading to lipid, protein and deoxyribonucleic acid (DNA) damage, cellular dysfunction and ultimately, fetal death [18].

The adipose tissue actsin a similar way to active endocrine tissuewell as an energy storage site. In addition, it creates adipokines and leptin that impact the immune response [25]. Visceral adipose tissue (VAT) appears to be more pathogenic than the subcutaneous depots do due to adipocyte hypertrophy, leading to local compression and hypoxia, which results in oxidative stress, cell apoptosis and necrosis [26].

In this way, the immune cells are activated, eliciting both a local and systemic low-grade inflammatory statewhich is characterized by moderately elevated levels of C-reactive protein (CRP) and pro-inflammatory cytokines such as TNF-α and IL-6, as well as a slightly increased number of pro-inflammatory neutrophils and mast cells and a decreased number of anti-inflammatory natural killer T cells and eosinophils. Therefore, it is not surprising that visceral adiposity has been associated with a higher risk of severe COVID-19 cases [27]. Cell membrane cholesterol plays an important role in viral infections, constantly interacting with high-density lipoproteincholesterol (HDL-C) and low-density lipoproteincholesterol (LDL-C). Low serum lipid levels in severe COVID-19 cases are due to an acute inflammatory response. In addition, HDL-C, LDL-C and triglyceride (TG) levels transiently decreased at the time of the COVID-19 diagnosis and later, they returned to the pre-infection levels two months after the SARS-CoV-2 infection. HDL is of particular importance in viral infections, especially SARS-CoV-2 infection. HDL consumption has been shown to impair its antiviral activity. Lipid rafts, which are cholesterol-rich microdomains on the host cell membranes, play a vital role in viral entry and budding. LDL promotes lipid raft formation, and it is possible that HDL depletes the cholesterol in the lipid rafts through cholesterol efflux from the cells. The depletion of cell membrane cholesterol reduced the risk of anSARS-CoV-2 infection by reducing the turnover of ACE2 and furin protease in the lipid rafts. In addition, scavenger receptor protein-B1 (SR-B1), which is an HDL receptor, has been shown to facilitate the ACE2-dependent entry of SARS-CoV-2. Lower HDL-C concentrations promote SRB1-mediated SARS-CoV-2 infections, whereas higher HDL-C concentrations inhibit SARS-CoV-2 infections. Apo-A1, an important component of HDL, has been shown to inhibit virus fusion and its entry into the host cells. Taken together, these data support our findings that increased serum HDL-C levels may be protective against an SARS-CoV-2 infection [28].

The discharge of the free radicals of oxygen such ashydrogen peroxide and superoxide anion by the polymorphonuclear cells and neutrophils is influenced by an increase in the amount of leptin. Leptin can also influence neutrophil migration by activating p38mitogen-activated protein kinase (MAPK) and extracellular signal-regulated kinase (Src) and increasing tumor necrosis factor-alpha (TNF-a) production by monocytes, while decreasing the chemotaxis process by blocking interleukin-8 (IL-8) [29]. As it is a member of the MAPK signaling family, p38 is largely associated with cellular stress responses and apoptosis. Anincreased amount of leptin causes an increase in the neutrophilic lung inflammation, and SARS-CoV-2 can cause critical ARDS [30].

Adipose tissue macrophages are referred to as classically activated (M1) macrophages. They release cytokines such as IL-1β, IL-6 and TNFα, creating a pro-inflammatory environment that blocks adipocyte insulin action, contributing to the development of IR and type 2 diabetes mellitus. In lean individuals, the macrophages are in an alternatively activated (M2) state. M2 macrophages are involved in wound healing and immunoregulation. Wound-healing macrophages play a major role in tissue repair and homoeostasis, while immunoregulatory macrophages produce IL-10, an anti-inflammatory cytokine, which may protect against inflammation. The functional role of Tcell accumulation has recently been characterized in adipose tissue. Cytotoxic Tcells are effector Tcells and have been implicated in macrophage differentiation, activation and migration [31,32]. These two hormones are crucial in the development of severe COVID-19 infections in people with inflammation because they are disrupted in obesity. Leptin and insulin resistance affect Tcell function in a reduced Tcell response to infection [33].

Macrophages in an activated state cause the death of the endothelial cells by the process of apoptosis or the secretion of proteolytic enzymes. The apoptotic effect of oxidized low-density lipoproteins (Ox-LDL) on endothelial cells can be attributed to the oxidation products of phosphatidylcholine or oxysterol. Under conditions of hypoxia, the oxidation of lipids occurs, especially polyunsaturated fatty acids (PUFA) and cholesterol. Oxysterols as cholesterol oxidation products are a diagnostic biomarker of oxidative stress [34]. The increased concentration of LDL particles in the bloodstream causes the person to become susceptible to a modification by the reactive oxygen and nitrogen particles, which are products of normal cellular metabolism. Endothelial cells, smooth muscle cells and macrophages are the main source of oxidizing particles for the oxidative modification of macromolecules in the subendothelium. In the early phase, mild oxidation leads to the formation of minimally modified LDL in the subendothelial space as a result of the peroxidation of unsaturated fatty acids in phospholipids and the oxidation of lecithin. Modified LDL particles are proinflammatory because they stimulate the production of signaling molecules and growth factors (vascular adhesion molecule-1, VCAM-1, monocyte chemotactic protein-1, MCP-1, and monocyte colony-stimulating factor, M-CSF) whichinfluence the movement of circulating monocytes and Tlymphocytes and their infiltration into the subendothelium of the blood vessels and inflammation. There is also the proliferation and differentiation of the monocytes into macrophages [30].

Further oxidation leads to the modification of lysine residues on apoB-100, which are crucial for the recognition of LDL by specific receptors. This leads to a decreased affinity of LDL particles for the LDL receptors. Over time, the LDL particles are so modified (glycosylated, ox-LDL or in the form of so-called advanced glycosylation end products—Advanced Glycosylation Endproducts, AGE particles) that they can no longer be introduced to the macrophages via the LDLreceptor, but rather, this occurs via the scavenger receptors. The characteristic of this receptor pathway is that there is no downstream regulation of the receptor, and no blockade of intracellular cholesterol synthesis by the inhibition of the enzyme HMG-CoA reductase. With the uncontrolled intake of ox-LDL, the macrophages accumulate cholesterol ester and lipid peroxides, and they pass into the so-called foamy cells [32].

Oxidatively modified LDL particles (Oh-LDL) can also be created by direct oxidation, which mainly takes place on unsaturated fatty acids under the influence of superoxide and peroxynitrite radicals, or by the transfer of already oxidized cellular lipids to low-density lipoproteins (LDL) [35]. In doing so, lipid peroxides are formed, which enter into a new chain reaction, as well as conjugated dienes and aldehyde products. These changes are preceded by a “hesitation period” of aldehyde oxidation, i.e., a latent period in which the antioxidants in LDL are consumed, after which the oxidation of the fatty acids begins. Degraded LDL simultaneously becomes a ligand for the receptors/catchers of the macrophages, and in this way monocytes are attracted to the site of the lesion, “take” lipoproteins and gradually turn into foam cells. When the LDL deposits exceed a critical threshold, the excessive activation of complement and macrophages creates the conditions for chronic inflammation [36]. Proinflammatory oxidatively modified LDL particles inhibit endothelial nitric oxide synthase (eNOS), leading to vasoconstriction by reducing the amount of available nitric oxide (NO). They damage the endothelial cells, and this leads to the apoptosis and necrosis of the vascular cells, all of which leads to the “leakage” of cellular lipids and lysosomal enzymes. There is a proliferation of macrophages and smooth muscle cells, the synthesis and secretion of numerous growth factors and pro-inflammatory cytokines from the surrounding cells, and the aggregation of the platelets. Additionally, these changed particles affect the retention of the macrophages in the arterial wall, inhibiting their mobility. The production of free radicals that intensify the oxidation of LDL is also increased. The cytotoxic effect leads to an increase in the concentration of calcium in the endothelial cells, and this process starts endothelial dysfunction. The foamy cells decay over time, and the lipid core of the atheromatous plaque is formed from the cellular detritus [33].

Specific arterial sites, such as forks and bends, lead to the accumulation of excess circulating LDL, endothelial damage at these sites, and changes in the blood flow. Specific molecules responsible for the adhesion, migration and accumulation of monocytes and Tlymphocytes are expressed in these places in the endothelium. Changes in blood flow alter the expression of the genes that provide information for the synthesis of adhesion molecules. Changes in the blood flow determine the location of the damage in the artery wall [34].

The inflammation process occurs in the intima of the blood vessels, and this is followed by the accumulation of macrophages, i.e., foam cells and T lymphocytes, which create a predisposition for long-term inflammation which manifests as a long-term COVID effect [37].

## 3. COVID-19 Diseases and Newborn

The initial metabolic response of the newborn to the lack of oxygen in the tissues is the activation of anaerobic glycolysis in the tissues. This process marks the consumption of the newborn child’s otherwise limited glucose reserves. As a result of the metabolic block, the concentration of lactate increases. Ketone bodies are three compounds (acetone, acetoacetic acid and beta-hydroxybutyric acid) that are produced as byproducts of the use of fatty acids for energy production in the liver and kidneys [38]. Ketone bodies are used as a source of energy in the heart and brain, and they are a vital source of energy for the brain during asphyxia. A change in the redox state of the cell in conditions of asphyxia, due to acidosis and the possible production of free radicals, can cause the cross-linking of membrane proteins by the formation of disulfide (S-S) bonds [39].

Lipid peroxidation, especially in the nerve cells, is catalyzed by iron that is released from hemoglobin, transferrin and ferritin, the release of which is induced by tissue acidosis and free oxygen radicals. Asphyxia increases the production of reactive oxygen metabolites, especially when the nerve cells are damaged [40]. Mitochondrial damage is a central event in a cell that is affected by ischemia as a consequence of asphyxia in severe respiratory distress caused by a COVID-19 infection in a newborn infant. The infant’s initial metabolic response to tissue oxygen deprivation caused by erythrocyte hemolysis during an SARS-CoV-2 infection, resulting in asphyxia, is the activation of anaerobic tissue glycolysis [41]. This process marks the consumption of the newborn’s otherwise limited glucose reserves [41]. Due to acidosis, the release of iron ions from ferritin, transferrin and hemoglobin is favored. Free ferrous iron (Fe++), which is has been found to bea chelator of certain precipitates, can form free radicals. Mitochondrial damage is a central event in a cell affected by ischemia as a consequence of asphyxia in severe respiratory distress caused by aCOVID-19 infection in a newborn infant [42].

The type of mitochondrial damage determines the type of cell death (necrosis or apoptosis). If the damage is intense, it quickly leads to the shutdown of mitochondrial function, the loss of adenosine triphosphate (ATP) production, the rupture of the nuclear and cytoplasmic membrane, which leads to necrosis, uncontrolled cell death (which occurs during hypoxia/ischemia) [43]. Apoptosis refers to controlled cell death, and this occurs in the reperfusion-reoxygenation phase. Necroptosis is a programmed necrosis or inflammatory cell disease. However, damaged mitochondria send a signal for the apoptotic program (by activating Cytochrome c or another intramitochondrial protein such as caspase, whichfragments the deoxyribonucleic acid (DNA) [44]. Apoptosis can be much more pronounced in the immature brain. Recent studies show that in the immature brain, in regions of the cerebral cortex and basal ganglia, there are a greater number of apoptotic cells than there are necrotic cells overa period that is longer than seven days after the hypoxic damage. During hypoxia, many other, different pathways are formed, including intermediates in the synthesis of prostaglandins during the activity of the enzyme cyclooxygenase [45]. Antioxidant molecules (AO) aim to prevent the formation of free radicals in the cell. They include three enzymes: superoxide dismutase (SOD), catalase (CAT) and glutathione peroxidase (GPx) whichdismutate •O_2_ and break down reactive hydrogen peroxide (H_2_O_2_) and hydroperoxides into “harmless” molecules [46].

The AO group includes transferrin and ceruloplasmin, which bind iron or copper, and consequently, prevent the formation of free radicals, as well as ascorbic acid, alpha tocopherol (Vitamin E). The AO is also produced by de novo enzymes that repair the damage to the biomolecules caused by the action of free radicals and repair the damage to the cell membranes. They have multiple roles: they recognize, break down and remove the oxidized or damaged proteins, DNA or lipids in order to prevent their accumulation, which can be toxic to tissues. They include, among others, DNA enzyme repair systems (polymerases, glycosylases and nucleases), as well as proteolytic enzymes (proteinases, proteases and peptidases), which are localized both in the cytosol and in the mitochondria of the mammalian cells. The AO of the fourth line of protection is the last line of defense that represents an adaptation in which the signals for the production and reactions of free radicals trigger the creation and transfer of specific antioxidants to the site of action. Signals arising from the formed radicals trigger the formation and transport of adequate AO to the site of action [47].

The disequilibrium between the production of reactive oxygen (ROS) and nitrogen (RNS) species and their elimination by protective mechanisms leads to oxidative stress. Mitochondria are the main source of ROS as they are by-products of the electron transport chain. Thus, the mitochondria greatly contribute to oxidative stress, a condition in which the production of free oxygen radicals exceeds their elimination. Lipid peroxidation affects not only the lipids of the cell membranes, but also the lipids in circulation, and there arereduced values of lipids and triglycerides in severe COVID-19 cases among pregnant women and newborns. Incomplete or transient hypoxia/ischemia does not immediately lead to cell death.

During hypoxia/ischemia, there is a prolonged depolarization of the membrane. This depolarization in some neuronal populations leads to the release of excitatory amino acids, among which glutamic and aspartic acids are the most significant ones. Glutamic acid is the most excitatory amino acid in the brain. It is cytotoxic in neuronal and cerebellar cells. Neuronal cells are activated to secrete glutamic acid by the entry of calcium into the cell and todepolarize in hypoxia. The fall in ATP leads to the maintenance of an increased concentration of glutamic acid, which further leads to the prolonged stimulation of receptors. The activation of these receptors leads to the entry of calcium into neurons with a detrimental effect on cell survival [48]. When the energy production is reduced, the ATP-dependent calcium-sodium pump cannot pump all of the calcium out of the cell. Proteases start the degradation of the cytoplasm, and endonucleases start the degradation of the DNA cleavage. This selective vulnerability (especially of the basal ganglia, brainstem and sensorimotor cortex) is probably more dependent on the intrinsic properties ofthe neurons (i.e., the excitatory receptors and reabsorption pumps) than it is on the circulation and redistribution of the cerebral flow. In addition, post-hypoxia states, ischemia, reperfusion and reoxygenation can induce the production of large amounts of nitric oxide (NO) in the neonatal brain tissue and cerebral circulation [43].

**Figure 2:**
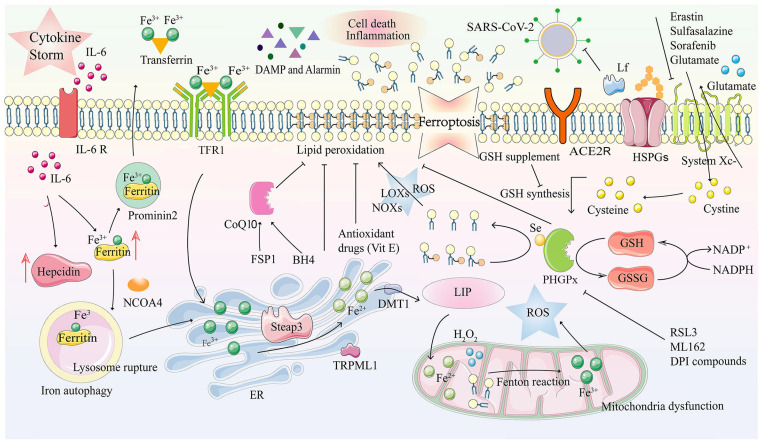
Ferroptosis and its mechanism in COVID-19. Ferroptosis is a kind of programmed cell death characterized by an imbalance in intracellular iron metabolism or a distortion of the glutathione peroxidation pathway. The transferrin receptor recognizes excess transferrin carrying Fe^3+^, and it enters cells through endocytosis after an SARS-CoV-2 infection. Metal reductase Steap3 reduces the Fe ions from trivalent to divalent, while the iron channels DMT1 and TRPML1 in the endosome membrane transport Fe^2+^ to the cytoplasm, which is accompanied by iron accumulation. In the case of intracellular iron overload, chemical substances in the mitochondrial electron transfer chain react with H_2_O_2_, Fe^2+^ and lipids, together, inducing the Fenton reaction, which produces large amounts of ROS. Due to GPX4 depletion and iron overload in LIP, lipid, nucleic acid and protein peroxidation results in cell membrane damage due to oxidative stress and ferroptosis. DAMPs and Alarmin (HMGB1, IL-33, TNF) are released, eventually aggravating cell death and inflammation. Tf and prominin 2 can effectively excrete the iron from the cells and inhibit ferroptosis. ACE2R, Angiotensin-converting enzyme 2 receptor; ALOXs, arachidonate lipoxygenases; BH4, tetrahydrobiopterin; CoQ10, coenzyme Q10; DAMP, damage-associated molecular patterns; DFO, deferoxamine; DMT1, divalent metal transporter 1; DPI, diphenyleneiodonium; ER, endoplasmic reticulum; FSP1, ferroptosis suppressor protein 1; GPX4, glutathione peroxidase 4; GSH, glutathione; GSSG, oxidized GSH; HMGB1, highmobilitygroupbox-1protein; HSPGs, heparan sulfate proteoglycans; H_2_O_2_, hydrogen peroxide; IL, interleukin; Lf, lactoferrin; LIP, labile iron pool; LOXs, lysyl oxidases; NADP^+^, nicotinamide adenine dinucleotide phosphate; NADPH, nicotinamide adenine dinucleotide phosphate; NCOA4, nuclear receptor coactivator 4; NOXs, NADPH oxidases; PLOOH, phospholipid hydroperoxide; PUFAs, polyunsaturated fatty acids; ROS, reactive oxygen species; RSL3, 1S,3R-RSL3; Se, selenocysteine; Steap3, six-transmembrane epithelial antigen of prostate 3; TFR1, transferrin receptors 1; TNF, tumor necrosis factor; TRPML1, transient receptor potential mucolipin 1; VitE, vitamin E. Reproduced from [49] (Sun C et al., 2020).

Ferroptosis is a new type of cell death that has been discovered in recent years, and it is usually accompanied by a massive iron accumulation and lipid peroxidation during the cell death process. Ferroptosis-inducing factors can directly or indirectly affect glutathione peroxidase through various pathways, resulting in a decrease in the antioxidant capacity and accumulation of lipid reactive oxygen species (ROS) in the cells, ultimately leading to oxidative cell death. The mechanism of ferroptosis of the COVID-19 disease can be explained by intracellular iron overload, chemical substances in the mitochondrial electron transfer chain reaction with H_2_O_2_, Fe++ and lipids, which produces large amounts of reactive oxygen species (ROS). Due to the glutathione peroxidase 4 (GPX4) depletion and iron overload in the labile iron pool (LIP), lipid, nucleic acid and protein peroxidation results in cell membrane damage due to oxidative stress and ferroptosis. Lipid peroxidation is an oxidative damage that affects the cell membranes, i.e., the lipids of the membrane lipoproteins of the newborn brain. The fact that most of the cholesterol is found in myelin indicates the fact that cholesterol is important for the maturation of the brain cells [49] (Figure 2).

## 4. Complications of COVID-19 Infections

The clinical characteristics of the consequences of anSARS-CoV-2 infection are different. About 20% of the adults had symptoms related to developing long-term complications of the virus [50].

The most common symptoms havea respiratory origin, although some of the patients feel tired for a long period of time. Previous studies have shown a number of potential late complications that are possible for a COVID-19 infection; these include pulmonary fibrosis, venous thromboembolism (VTE), arterial thromboses, cardiac thrombosis and inflammation, stroke, “brain fog,” dermatologic complications, and general mood dysfunctions and hair loss [51]. The ways that lead to long-term COVID-19 infection complications include endothelial injury, immune system dysregulation and hypercoagulability often leading to thrombosis. The most likely mechanism is an abundant cytokine syndrome that leads to cell damage. It is assumed that the activation of macrophages and T lymphocytes in the intima of the blood vessels creates the basis for the formation of arterial plaque. Additionally, the creation of autoantibodies against different cells of the body and the disruption of the autonomic nervous system leads to a post-viral disease [52]. A lot of pro-inflammatory cytokines and chemokines such as IL-6, IL1B, IFNγ, IP10, and MCP1 IL2, IL7, IL10, GCSF, MIP1A and TNF-α have been observed in patients infected with SARS-CoV-2. The main factor in the development of cytokine release syndrome (CRS) is IL-6. The uncontrolled increase in the IL-6 level causes an over-immune response and prolonged infection symptoms [53].

The patients with COVID-19 have significant dyslipidemia, which is closely related to cardiovascular disease. The concentration of HDL, LDL-C, and total cholesterol (TC) in the COVID-19 patients was significantly reduced. HDL is significantly decreased in the critically ill patients, while LDL and TC are decreased, but they were still dominant 53 of them, indicating an increased risk of cardiovascular disease in the COVID-19 patients [54].

The inflammatory nature of an SARS-CoV-2 infection during pregnancy may cause adverse obstetric and neonatal events [55]. Exposure to intrauterine inflammation and placental changes can lead to long-term, multisystem defects in exposed neonates. There is more and more evidence that an intrauterine infection with the SARS-CoV-2 virus leaves long-term consequences on the fetal brain. Neurological sequelae manifested in neonates are in the form of drowsiness, apneic crises, cyanosis crises, neonatal seizures and epilepsy at an early age [56,57,58].

## 5. Conclusions

COVID-19 is not only an infectious disease caused by the SARS-CoV-2 virus, but it is also a metabolic disease. Lipid peroxidation mediated by asphyxia caused by the COVID-19 disease can cause the death of a newborn and pregnant women as well as short-term and long-term sequelae.

## Data Availability

All data are available in the archives (database) medline, PubMed.
